# Multilevel and Multiscale Feature Aggregation in Deep Networks for Facial Constitution Classification

**DOI:** 10.1155/2019/1258782

**Published:** 2019-12-20

**Authors:** Er-Yang Huan, Gui-Hua Wen

**Affiliations:** School of Computer Science and Engineering, South China University of Technology, Guangzhou 510006, China

## Abstract

Constitution classification is the basis and core content of TCM constitution research. In order to improve the accuracy of constitution classification, this paper proposes a multilevel and multiscale features aggregation method within the convolutional neural network, which consists of four steps. First, it uses the pretrained VGG16 as the basic network and then refines the network structure through supervised feature learning so as to capture local image features. Second, it extracts the image features of different layers from the fine-tuned VGG16 model, which are then dimensionally reduced by principal component analysis (PCA). Third, it uses another pretrained NASNetMobile network for supervised feature learning, where the previous layer features of the global average pooling layer are outputted. Similarly, these features are dimensionally reduced by PCA and then are fused with the features of different layers in VGG16 after the PCA. Finally, all features are aggregated with the fully connected layers of the fine-tuned VGG16, and then the constitution classification is performed. The conducted experiments show that using the multilevel and multiscale feature aggregation is very effective in the constitution classification, and the accuracy on the test dataset reaches 69.61%.

## 1. Introduction

The constitution in TCM (traditional Chinese medicine) refers to the relatively stable body traits of the individual to the internal and external environment of the body. It is a morphological structural psychological state and physiological function formed on the basis of congenital inheritance, which is a system concept formed by combining the Chinese medical discourse on human physique phenomena and the understanding of physique in many disciplines and the purpose of medical research [1]. Constitution phenomenon is an important manifestation of human life phenomenon. It has the characteristics of individual difference group homogeneity and relative stability and dynamic variability [2, 3].

Constitution classification is the basis and core content of TCM constitution research. The purpose is to standardize human constitution categories, and then to give different personalized conditioning options for different constitution types. Therefore, it is especially important for specific people to accurately identify their constitution categories. The commonly used identification method is based on the questionnaire. All questions are answered and then scored to determine the type of constitution according to the national standard “classification and determination of Chinese medicine constitution” [4]. This method has the following shortcomings [5, 6]:

1. It is influenced by the subjective factors. Individuals are not very familiar with some problems, so that it is difficult for them to accurately choose answers. Second, individuals have concerns about some private issues and are reluctant to choose real answers.
2. The number of questions to be answered is relatively large. It takes long time to answer these questions, easily making individuals lose patience to answer the problems located at the end of the questionnaire. These problems are often randomly selected, which will inevitably affect the correct judgment of the constitution.
3. The calculation method of scores is much complex, so that constitution types of many people cannot be accurately calculated.

In order to solve these problems, many new methods have been proposed [7–9]. For example, Su et al. [7] studied the acoustic characteristics of eight different constitutions and applied them to constitution recognition. Hou et al. [8] extracted the color and texture features of the face and then classified the body constitution. Lu et al. [9] extracted the color and texture features of the tongue and performed feature fusion. These methods use traditional feature representations such as color, texture, histogram of oriented gradient (HOG), and so on. However, these methods extracted manually designed features focusing on the local pattern of the object while ignoring the semantic information, so that these features usually have limited performance. Recently, many scholars have applied machine learning algorithms to TCM constitution recognition [10, 11]. For example, Wang and Bai [10] applied the BP neural network to pulse diagnosis to classify the type of constitution and then demonstrated its rationality and superiority. Zhang et al. [11] proposed a dynamic classification model algorithm based on relevance, constructing feature indicators on face skin and then used the improved decision tree and the fuzzy naive Bayesian algorithm to classify the constitution. Moreover, with the rapid spread of CNN, many visual recognition tasks have achieved outstanding achievements, such as image classification [12, 13], image segmentation [14, 15], object detection [16, 17], and scene recognition [18, 19]. Instead of manually designing visual features, CNN provides an end-to-end feature learning framework that automatically learns deep representations of images from a global view. Some researchers have also applied CNN to constitution recognition. Hu et al. [20] applied the convolution neural network to the pulse diagnosis. In the case of feature ambiguity, the proposed method is superior to other well-known methods. Li et al. [21] used the convolution neural network to extract the features of the pulse and then classify the body constitution. The experimental results show that this method can obtain high accuracy. Huan et al. [22] proposed a constitution recognition algorithm based on the convolutional neural network, which trained a convolutional neural network model for constitution recognition on face data. Li et al. [23] proposed a constitution recognition algorithm based on the deep neural network, which first detected the tongue image and then determined the body constitution type. Hu et al. [24] proposed a classification algorithm based on the tongue, which uses the Inception v3 model for constitution classification. Zhou et al. [25] also proposed a physique classification algorithm based on the tongue. This method first cuts the tongue and then classifies its type, but it has only three categories. Ma et al. [26] proposed a complex perception-based algorithm for constitution recognition, whose dataset is tongue images. These methods are designed to predict the constitution categories by learning high-level semantic features through a hierarchical architecture. As the depth of the network increases, more abstract and higher level features can be extracted. However, the above methods are highly concerned with high-level semantic features, while intermediate features are ignored in the final classification. Moreover, the CNN-based methods typically rely on large-scale, manually tagged training datasets, such as the ImageNet [27]. However, in the field of Chinese medicine, it is very expensive to label large amounts of data by Chinese medicine experts, which could be obtained in the short time. Training a convolutional neural network model from scratch is not easy, and it takes a long time, even requires some patience and expertise in training neural networks [28].

**Figure 1:**
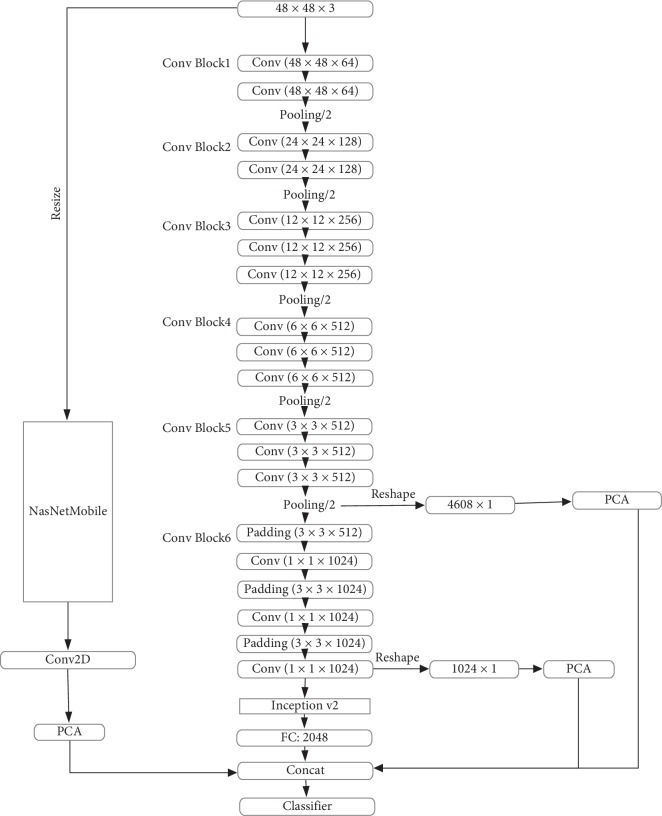
Overview of our proposed algorithm.

Considering the above challenges, in this paper, we propose a constitution classification algorithm based on pretrained convolutional neural networks for the aggregation of multilevel and multiscale features.  Figure 1 shows an overview of the proposed algorithm. Our algorithm is divided into four steps, namely, network training, feature extraction and dimension reduction, feature aggregation, and constitution classification. First, the VGG16 [29] and NASNetMobile [30] network structures are fine-tuned by the transfer learning method. Second, the features of the different layers in the fine-tuned VGG16 are extracted and the PCA is performed to these features. Third, the previous layer features of the global average pooling layer in NASNetMobile are extracted and then performed by PCA. These dimensionally reduced features are aggregated with the fully connected layer features in the fine-tuned VGG16 so as to obtain aggregated features. In the final stage, the aggregated features are input into the classifier to perform the constitution classification.

**Figure 2:**
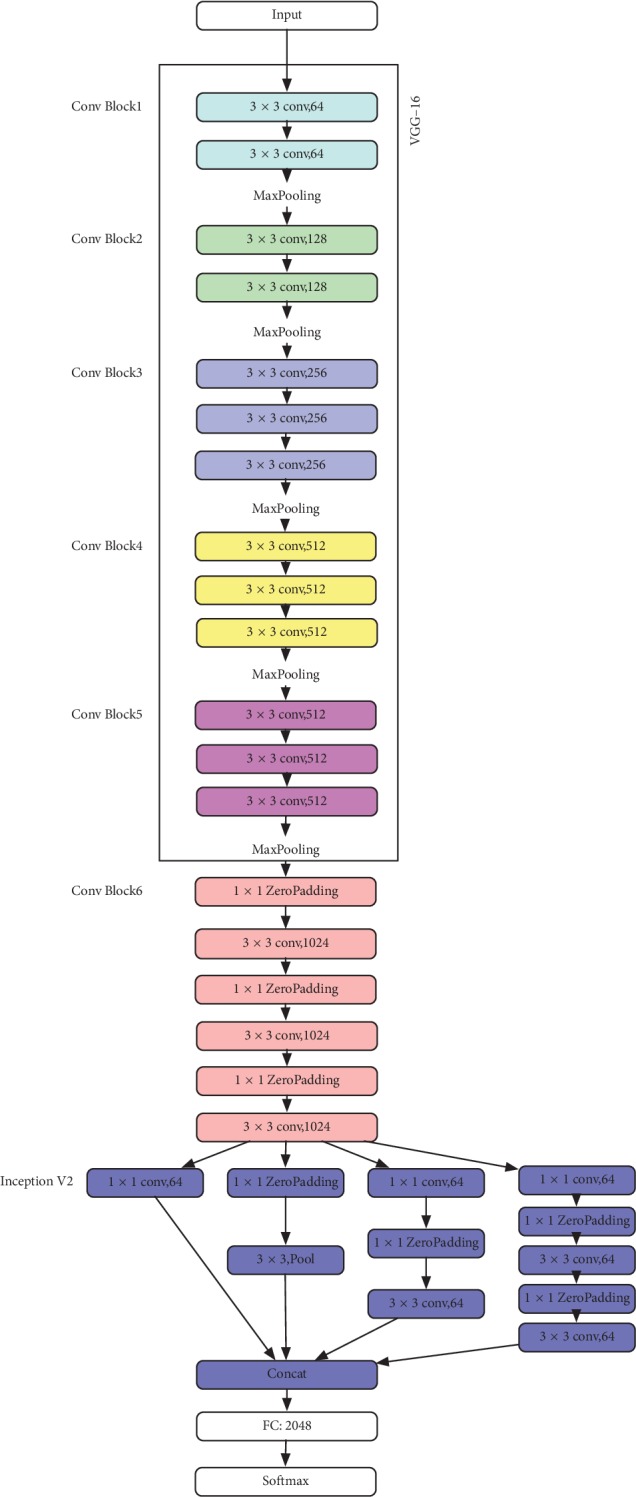
The architecture of CNN improved from VGG16.

In this article, we make the following innovative contributions:

1. An improved version of the VGG16 network, called VGG-CI, is proposed and shown in Figure 2, which added two modules: Conv Block and Inception V2 [31]. The Conv Block module contains three cascaded convolution modules, while the Inception V2 module increases the ability to represent features. By adding the Conv Block and Inception V2 modules, the depth and width of the network are further increased to improve the classification effect.
2. A fusion method of multiview features is proposed. First, the output of the different layers in the VGG-CI network represents features of different levels of abstraction. Based on the VGG-CI network, we extract the features of different layers, perform PCA to them, and then aggregate them with the fully connected layer to obtain the output V1. In order to continue to increase the feature representation capability, the NASNetMobile model is used to extract features from another view. By obtaining the previous layer features of the global average pooling layer, PCA also applied these features, which are then aggregated with V1.
3. A large and high-quality database of clinical facial images is constructed, which can nicely support the research of facial constitution classification algorithm. At present, no institute provides a large number of high-quality clinical databases for facial constitution classification.

The structure of the paper is as follows: in Section 2, we present the method proposed. In Section 3, lots of experiments are conducted to validate the proposed method. The conclusion is presented in Section 4.

## 2. Proposed Approach

The overall architecture we propose is shown in Figure 1. In this section, we will detail our approach. First, the network for feature extraction is introduced. Second, the aggregation method of multilevel and multiscale features is introduced. Finally, the constitution classification algorithm is introduced.

### 2.1. Feature Extraction Network

In the case of limited medical image dataset, it is likely that the convolutional neural network could not learn the features of the image well during the training process. Because the pretrained CNN network was trained on millions of different images, it contains powerful generic feature extraction filters. In order to extract the optimized features from the image, we used the well-verified CNN architecture, namely, VGG16 and NASNetMobile. These networks contain the max pooling and cascaded convolution layers. The total number of layers depends on each network. VGG is known for its elegance and simplicity, while it has near state-of-the-art results in image classification and good versatility. The VGG16 is the runner-up in the 2014 large-scale visual recognition challenge. In our work, we used a pretrained VGG16 network with 16 weight layers, 13 convolution layers, and 3 fully connected layers. Then, we changed the top layer as we needed, by adding the Conv Block and Inception V2 modules. The Conv Block contains 3 ZeroPadding layers and 3 convolution layers. The Inception V2 module contains a 1 × 1 convolutional layer that reduces the amount of computation while increasing the network width. By adding the Conv Block and Inception V2 modules, the network has a stronger feature representation capability for the better classification. The network structure is shown in Figure 2. The image size entered in this network is 48 × 48.

### 2.2. Multilevel and Multiscale Feature Aggregation

**Figure 3:**
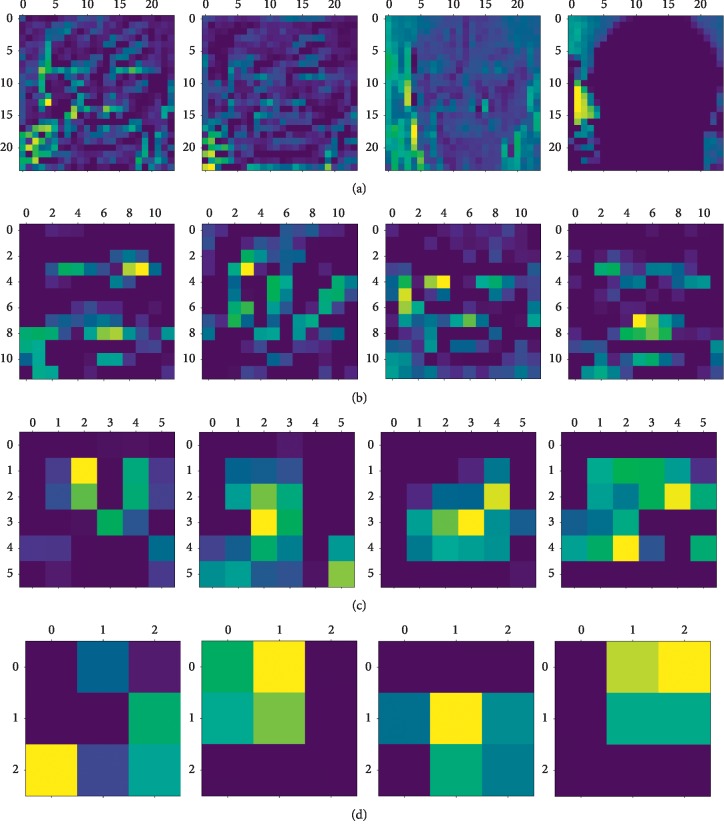
The feature maps of different layers: (a) layer 3, (b) layer 6, (c) layer 10, and (d) layer 14.

The pretrained CNN can be considered as a feature extractor. A single CNN model can extract features of different layers for the given input size. As shown in Figure 2, the network contains six Conv Block modules, one Inception V2 module, and one fully connected layer. We train the network on the training dataset and then extract the features of different layers. First, for a given 48 × 48 image, the feature map sizes of the 3^rd^, 6^th^, 10^th^, 14^th^, and 24^th^ layers are 24 × 24, 12 × 12, 6 × 6, 3 × 3, and 1 × 1, respectively. This article shows the feature map of the 3^rd^, 6^th^, 10^th^, and 14^th^ layers, as shown in Figure 3, respectively. In this paper, we use the information of the middle layers, namely, the 14^th^ and 24^th^ layers are selected. However, the number of features for the 14^th^ and 24^th^ layers is (3, 3, 512) and (1, 1, 1024), respectively. In order to be able to carry out further feature dimensionality reduction, it is necessary to flat the dimensions of the 14^th^ and 24^th^ layers, leading to 4608 and 1024 features, respectively. Subsequently, PCA is applied to these features. It can be seen from Figure 3 that in the lower layer, the features are more and complicated, while features in the higher layers are simple, only the distinguishing features are needed. The abstraction levels of different layer features are different. Obviously, the abstraction leads to information loss, so that the features of different layers should be merged to compensate for the loss.

At the same time, we use the pretrained NASNetMobile network for feature extraction. The network consists of two modules: normal cell and reduction cell. The stacking of these two modules is then applied to form the whole network. In this network, we resized the image to 224 × 224 and then inputted it into the network. After training, it needs to extract the previous layer features of the global average pooling layer, in which PCA is applied to perform dimensionality reduction.

Finally, we aggregate the features of the 14^th^ and 24^th^ layers, features of the previous layer of the global average pooling layer, and features of the fully connected layer as shown in Figure 2 to obtain the aggregated features. The aggregated features are then entered into the classifier.

### 2.3. Constitution Classification

In this step, we predict the type of constitution based on the multilevel and multiscale aggregated features. There are lots of pattern classification algorithms, such as support vector machine(SVM) [32], K nearest neighbor(KNN) [33], Bayesian classifier(NB) [34], decision tree(DT) [35], logistic regression(LR) [36], and random forest algorithm(RF) [37]. Ensemble learning improves the effectiveness of machine learning by combining several models. This method can provide the better prediction results than a single model. This article also uses ensemble learning methods for classification, such as XGBoost [38], LightGBM [39], and CatBoost [40]. In our experiments, we evaluated the classification effects of different classifiers.

## 3. Experiments

### 3.1. Dataset

The face data used in this paper have 21,150 pictures, which are obtained from the Chinese medicine clinic of the three hospitals, in which each facial image of the patient is assigned a constitution type by a professor of Chinese medicine. The identification of the constitution type is based on the national judgment criteria [41] for TCM constitution. Before collecting data, the standard is discussed by nearly ten medical experts. Some agreed with this standard. Some professors were partially in favor of the standard. Some professors have a negative attitude on this standard. We chose three professors who were in favor of this standard. This means that they reached the consensus (agreement of standard) to determine the type of body constitution. Subsequently, they were in different hospitals to judge the patient's body constitution according to the standard. In this way, the impact of experience can be reduced as much as possible. Besides, these professors are well known and their ages are close and the personal experience is not greatly different. Finally, the body constitution type of the patient in the same hospital is determined by the same medical professor. The entire dataset is determined by three Chinese medicine professors from three different hospitals according to the abovementioned standard.

**Figure 4:**
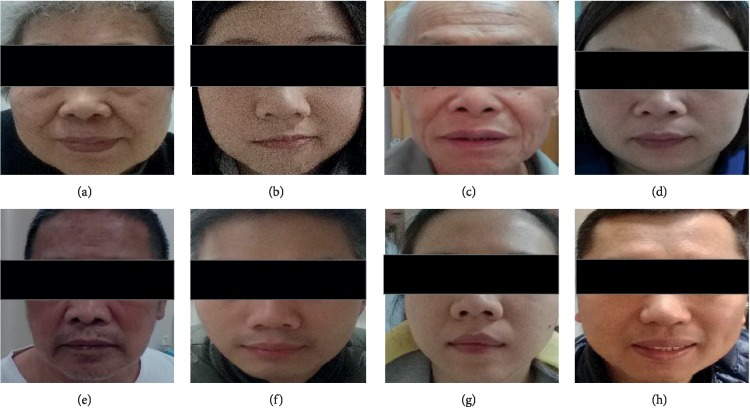
Examples in different constitution types: (a) Qi-deficiency, (b) Yin-deficiency, (c) Yang-deficiency, (d) phlegm-dampness, (e) dampness-heat, (f) Qi-depression, (g) blood-stasis, and (h) gentleness.

Therefore, all face images are taken by the same type of digital device and the patient's constitution type is specified by the doctor. The indoor environment is no sunshine, and lighting conditions are normal fluorescent lamps. In the face database, there are 8 kinds of constitution types, that is, gentleness, Qi-deficiency, Qi-depression, dampness-heat, phlegm-dampness, blood-stasis, Yang-deficiency, and Yin-deficiency. The number of samples with each constitutional type is given in Table 1. Samples with constitution types are shown in Figure 4. In the preprocessing process, the face detection algorithm is used to detect the acquired picture and the corresponding bounding box is obtained. Considering both time complexity and precision, this paper uses the OpenCV tool to complete the face detection. The test dataset used in this paper is the test dataset used in [22], and the training dataset does not overlap with the test dataset.

### 3.2. Data Augmentation

This article uses the data augmentation when training the VGG16 networks and NASNetMobile networks. In this paper, the width and height of each facial image are scaled proportionally and the image is zoomed in both length and width direction. This paper uses the Keras [42] tool to achieve data augmentation through the functions it contains. It just sets the values of width_shift_range, height_shift_range, and zoom_range in the ImageDataGenerator function. After data augmentation, it trains the network on these training samples through transfer learning.

### 3.3. Training Details

The tools used in this experiment are Keras, TensorFlow [43], Scikit-learn [44], and Scikit-image [45]. The GPU is NVIDIA GTX Titan X, the memory size is 12 GB, and the operating system is Ubuntu 14.04. The VGG16 and NASNetMobile networks are with the same setting. They are trained by the random gradient method. The learning rate is 0.0002, the momentum is set to 0.9, and the batch size is set to 30. In data augmentation processing, the values of width_shift_range, height_shift_range, and zoom_range are all set to 0.2.

### 3.4. Experimental Results and Discussion

In the previous works, many traditional feature extraction methods have been applied to perform the constitution recognition. In order to show the superiority of deep learning methods to traditional feature extraction methods, lots of experiments are conducted to make comparison with them. Traditional facial feature extraction methods include color, texture, histogram of oriented gradient, and so on. Here, the color feature is represented by the HSV space and the texture feature is represented by local binary patterns. The classifiers in this article are described in Section 2.3. The settings of the classifier are as follows: RBF is selected in the support vector machine. The learning rate of the CatBoost classifier is 0.05, and the depth is 6. The learning rate is 0.05 in the Xgboost classifier, and the max_depth is 6. In the LightGBM classifier, the learning rate is 0.009, the max_depth is 8, the lambda_l1 and lambda_l2 are set to 0.9, and the num_leaves is 90. CatBoost, Xgboost, and LightGBM are abbreviated as Catb, Xgb, and Lgb in our paper, respectively. In order to make comparison among different feature extraction methods through experiments, we use the same classifier.

It can be seen from Table 2 that under the premise of the same classifier, the classification effect based on the VGG16-CI network extraction feature is better than that based on the single HSV, LBP, and HOG features. On the contrary, under the same feature extraction method, the classification effects of different classifiers are compared. Based on the single HSV feature and the LBP feature, SVM has the best classification accuracy. Based on the single HOG feature, Random Forest classification has the best performance. Based on the features extracted by the VGG16-CI network, the LightGBM has the best classification effect. Overall, the VGG16-CI network is far better than other feature extraction methods. At the same time, the confusion matrix of each classifier is expressed as follows. It can be seen from Tables 3 and 4 that the selected classifier has a good classification effect on the Qi-deficiency and a poor classification effect on the gentleness. This is because the quality of gentleness is affected by the Qi-deficiency. It can be seen from Table 5 that the classifier has a good classification effect on the Yin-deficiency. The effect on the gentleness is poor which is also affected by the Yin-deficiency. It can be seen from Table 6 that the classifier has a good classification effect on the Qi-deficiency. The effect on the gentleness is poor which is also affected by the Qi-deficiency. It can be seen from Table 2 that the classification of LightGBM is the best, whose confusion matrix is presented as given in Table 6. Therefore, in the following experiments, LightGBM is selected as the classifier for constitution recognition.

In this paper, the VGG-CI model is obtained by transfer learning based on VGG16. The features of different layers of the VGG-CI model are extracted to classify the facial images. In more detail, the features of the 14^th^ layer, 24^th^ layer, and fully connected layer are selected. Subsequently, the PCA method is used to reduce the dimensionality of features of the 14^th^ layer and the 24^th^ layer, respectively. The number of merged features is 100. Each is then merged with the features of the fully connected layers. Finally, these merged features are applied to perform the classification, in which the classifier is the LightGBM. The classification results are shown in Table 7. It can be seen that the classification accuracy of the 14^th^ layer is very low, and the classification effect reaches 68.67% in the fully connected layer. At the same time, the features of the 14^th^ layer and the 24^th^ layer are dimensionally reduced by PCA, respectively, which is then applied to merge with the features of the fully connected layer. In such case, the classification effect is further improved.

On the contrary, we also use the transfer learning to further train the NASNetMobile network, extracting the features of the previous layer of the global average pooling layer. This layer is represented by Conv2D, whose features are flatted, and then PCA is applied to obtain fewer features. These features, the features of the 14^th^ layer after PCA, the features of the 24^th^ layer after PCA, and fully connected layers, are aggregated, and the classification accuracy reached 69.61%.

**Figure 5:**
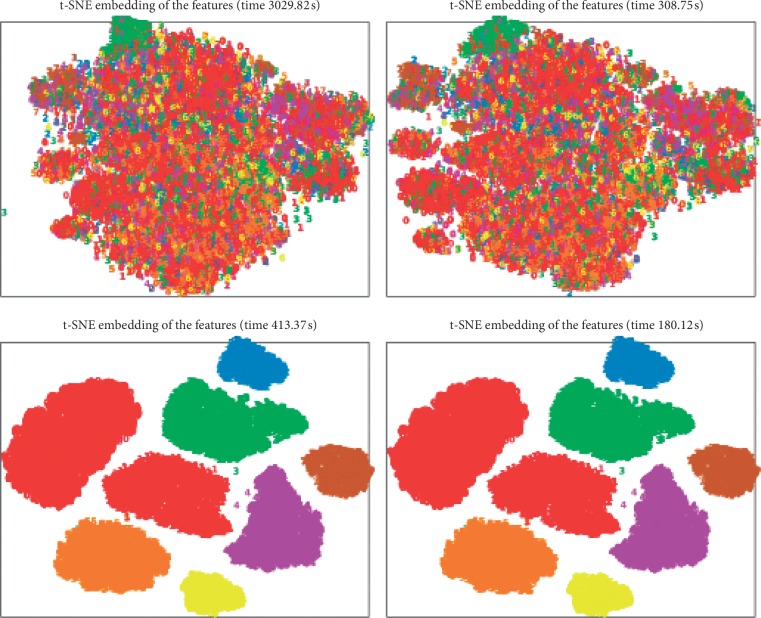
Visualization of the extracted features for the training data by t-SNE. The first row is for the 14^th^ layer, and the second row is for the 24^th^ layer. The left column is the original features, and the right column is the features after PCA.

In order to visually analyze the performance of our method, t-SNE is applied to display the extracted features form on the training dataset, as shown in Figure 5. It can be seen from Figure 5 that as to the features of the 14^th^ layer, the difference can be observed between the original features and dimensionally reduced features by PCA. It validates the usage of PCA. However, as to the 24^th^ layer features, there is no significant change after the PCA is used. This is because the original features have been good enough. This illustrates that PCA should be applied appropriately.

Now, there are some well-known deep learning networks, such as VGG19 [29], Inception v3 [31], ResNet [46], InceptionResnet [47], Xception [48], MobileNet v1 [49], DenseNet [50], EfficientNets [51],and NASNet [30]. In order to verify the classification effect of the proposed algorithm, we use the transfer learning to train these networks with the same facial training dataset. The classifier is also the LightGBM that has the same setting. The experimental results are shown in Table 8. It is easy to see that the algorithm this paper proposed works best, and the accuracy rate is 69.61%. Moreover, in the case of the same test dataset, the method proposed in this paper is compared with the method of [22]. The literature [22] aggregated the features extracted from convolutional neural networks with the traditional color feature. Its classification accuracy was 65.29%. The algorithm proposed in this paper adopts the multiscale and multilayer feature aggregation method, so that the better classification accuracy is obtained up to 69.61%. The confusion matrix of the proposed algorithm is shown in Table 9. It can be seen that the quality of the gentleness is still affected by the Qi-deficiency.

## 4. Conclusion and Future Work

In this paper, we propose a constitution classification algorithm based on a newly designed convolutional neural network, which makes full use of the information of different layers in the network. We also demonstrate the effectiveness of our approach by merging multilevel and multiscale features. Finally, the method proposed in this paper achieves the best results on the test set. In the future, we plan to improve our designed network by using the new learning method. At the same time, the impact of Qi-deficiency on gentleness should be considered.

## Figures and Tables

**Table 1:** The number of samples with different constitution type.

	Gentleness	Qi-deficiency	Yang-deficiency	Yin-deficiency	Phlegm-dampness	Dampness-heat	Blood-stasis	Qi-depression	Sum
Training dataset	1381	4806	1234	3489	3378	2973	1069	2820	21150
Testing dataset	57	75	60	75	75	75	41	75	533

**Table 2:** Classification results of different classifiers under different feature extraction.

	SVM (%)	RF (%)	KNN (%)	NB (%)	Softmax (%)	DT (%)	Catb (%)	Lgb (%)	Xgb (%)
HSV	64.91	62.47	23.26	17.82	17.63	59.66	27.20	26.45	55.91
LBP	64.91	60.78	24.02	14.82	16.14	56.85	58.54	23.45	57.79
HOG	19.14	60.78	25.7	16.32	14.07	57.22	34.52	32.65	59.29
VGG16-CI	67.17	66.04	66.79	66.60	67.72	64.72	66.42	68.67	67.35

**Table 3:** The confusion matrix of the SVM algorithm based on the HSV feature.

	Qi-deficiency	Yin-deficiency	Yang-deficiency	Phlegm-dampness	Dampness-heat	Qi-depression	Blood-stasis	Gentleness
Qi-deficiency	**75**	0	0	0	0	0	0	0
Yin-deficiency	14	**61**	0	0	0	0	0	0
Yang-deficiency	25	0	**35**	0	0	0	0	0
Phlegm-dampness	15	0	0	**60**	0	0	0	0
Dampness-heat	23	0	0	0	**52**	0	0	0
Qi-depression	44	0	0	0	0	**31**	0	0
Blood-stasis	9	0	0	0	0	0	**32**	0
Gentleness	57	0	0	0	0	0	0	**0**

**Table 4:** The confusion matrix of the SVM algorithm based on the LBP feature.

	Qi-deficiency	Yin-deficiency	Yang-deficiency	Phlegm-dampness	Dampness-heat	Qi-depression	Blood-stasis	Gentleness
Qi-deficiency	**75**	0	0	0	0	0	0	0
Yin-deficiency	14	**61**	0	0	0	0	0	0
Yang-deficiency	25	0	**35**	0	0	0	0	0
Phlegm-dampness	15	0	0	**60**	0	0	0	0
Dampness-heat	23	0	0	0	**52**	0	0	0
Qi-depression	44	0	0	0	0	**31**	0	0
Blood-stasis	9	0	0	0	0	0	**32**	0
Gentleness	57	0	0	0	0	0	0	**0**

**Table 5:** The confusion matrix of the random forest algorithm based on the HOG feature.

	Qi-deficiency	Yin-deficiency	Yang-deficiency	Phlegm-dampness	Dampness-heat	Qi-depression	Blood-stasis	Gentleness
Qi-deficiency	**44**	17	0	9	3	2	0	0
Yin-deficiency	5	**64**	0	4	1	1	0	0
Yang-deficiency	16	2	**35**	4	1	1	0	1
Phlegm-dampness	7	4	0	**63**	0	1	0	0
Dampness-heat	11	3	0	5	**53**	3	0	0
Qi-depression	29	5	0	7	1	**33**	0	0
Blood-stasis	5	1	0	2	1	0	**32**	0
Gentleness	27	10	0	12	5	3	0	**0**

**Table 6:** The confusion matrix of the LightGBM algorithm based on the VGG-CI feature.

	Qi-deficiency	Yin-deficiency	Yang-deficiency	Phlegm-dampness	Dampness-heat	Qi-depression	Blood-stasis	Gentleness
Qi-deficiency	**68**	3	1	1	2	0	0	0
Yin-deficiency	3	**66**	0	2	3	1	0	0
Yang-deficiency	15	3	**36**	1	4	0	1	0
Phlegm-dampness	7	2	0	**63**	1	1	0	1
Dampness-heat	13	0	3	1	**58**	0	0	0
Qi-depression	13	3	1	3	6	**41**	0	8
Blood-stasis	7	1	0	0	0	1	**32**	0
Gentleness	29	6	2	5	4	7	2	**2**

**Table 7:** Classification results of different layers.

Layer	Accuracy (%)
14^th^ layer	53.65
24^th^ layer	66.97
FC layer	68.67
(14^th^ layer + PCA) + FC	68.48
(24^th^ layer + PCA) + FC	68.85
(14^th^ layer + PCA) + (24^th^ layer + PCA) + FC	69.04
(14^th^ layer + PCA) + (24^th^ layer + PCA) + (Conv2D + PCA) + FC	69.61

**Table 8:** Classification results of different models.

Model	Accuracy (%)
VGG19 [29]	59.47
Inception v3 [31]	64.17
ResNet-50 [46]	65.47
InceptionResnet [47]	62.48
MobileNet v1 [49]	63.98
Xception [48]	64.54
DenseNet-121 [50]	62.47
DenseNet-169 [50]	63.04
DenseNet-201 [50]	64.16
NASNetMobile [30]	62.85
EfficientNetsB0 [51]	63.23
Lit. [22]	65.29
Our proposed method	69.61

**Table 9:** The confusion matrix of the algorithm proposed in this paper.

	Qi-deficiency	Yin-deficiency	Yang-deficiency	Phlegm-dampness	Dampness-heat	Qi-depression	Blood-stasis	Gentleness
Qi-deficiency	**68**	3	1	1	2	0	0	0
Yin-deficiency	4	**66**	0	1	3	1	0	0
Yang-deficiency	12	4	**36**	2	4	1	1	0
Phlegm-dampness	7	2	0	**63**	1	1	0	1
Dampness-heat	13	0	3	1	**58**	0	0	0
Qi-depression	7	3	2	3	6	**46**	0	8
Blood-stasis	6	1	0	0	0	2	**32**	0
Gentleness	26	6	2	5	4	10	2	**2**

## Data Availability

The TCM data used in this study can be obtained by contacting the corresponding author.
